# Nifedipine-Induced Changes in the Electrohysterogram of Preterm Contractions: Feasibility in Clinical Practice

**DOI:** 10.1155/2010/325635

**Published:** 2010-06-16

**Authors:** Maartje P. G. C. Vinken, C. Rabotti, M. Mischi, J. O. E. H. van Laar, S. G. Oei

**Affiliations:** ^1^Department of Obstetrics and Gynecology, Máxima Medical Centre, De Run 4600, 5504 DB Veldhoven, The Netherlands; ^2^Department of Electrical Engineering, Eindhoven University of Technology, Den Dolech 2, 5612 AZ Eindhoven, The Netherlands

## Abstract

*Objective*. Evaluating changes in the power spectral density (PSD) peak frequency of the electrohysterogram (EHG) caused by nifedipine in women with preterm contractions. *Methods*. Calculation of the PSD peak frequency in EHG contraction bursts at different times of nifedipine treatment in women in gestational age 24 to 32 weeks with contractions. *Results*. A significant (*P* < .05) decrease of PSD peak frequency between EHG signals measured before and 15 minutes after administration of nifedipine. A significant (*P* < .05) decrease in PSD peak frequency comparing signals recorded within 24 hours after administration of nifedipine to signals 1 day after tocolytic treatment. A higher average PSD peak frequency for patients delivering within 1 week than that for patients delivering after 1 week from nifedipine treatment (*P* > .05). *Conclusions*. EHG signal analysis has great potential for quantitative monitoring of uterine contractions. Treatment with nifedipine leads to a shift to lower PSD peak frequency in the EHG signal.

## 1. Introduction

Preterm birth is the leading cause of perinatal mortality and morbidity and accounts for approximately half of preterm births [1]. Although the management of threatened preterm labor by tocolytic therapy can prolong gestation [2], effectiveness of tocolytic agents depends on early introduction of therapy [1]. Some risk factors for preterm delivery have been identified, but available prediction methods exhibit poor diagnostic value for detecting preterm labor at an early stage [3–5].

When evaluating women with preterm contractions, the clinicians' daily dilemma is to differentiate between physiological uterine activity and contractions leading to preterm delivery. Monitoring uterine contractions may provide important prognostic information during pregnancy and parturition. No method is currently available for non-invasive monitoring and quantification of uterine contractions [3–5]. 

It is well established that uterine contractions are the result of the electrical activity propagation through the uterine muscle cells [6, 7]. The electrohysterogram (EHG) is a direct measure of the myometrium electrical activity and it can be measured noninvasively by electrodes positioned on the maternal abdomen [8–10]. The EHG is characterized by intermittent “bursts” of action potentials and each burst corresponds to a mechanical contraction of the uterus. The uterus is quiescent during pregnancy. However, as the delivery time approaches, the uterine electrical activity becomes increasingly synchronous [11]. 

Some typical changes have been observed in the EHG properties during preterm and term delivery [12–15]. In particular, the shifting of the EHG signal energy to higher frequency as delivery approaches is the most experienced phenomenon observed in the literature [11–14, 16]. In particular, the peak frequency, that is, the frequency corresponding to the maximum of the EHG signal power spectral density (PSD), is the EHG parameter that is most commonly tested [17]. 

As uterine preterm contractions are the most frequently recognized precursor of preterm delivery, inhibiting contractions (tocolysis) has been the focus of therapeutic approaches for prevention of preterm birth. 

As the delivery time approaches, the number of cell gap junctions and voltage-dependant calcium channels increases and form an electrical syncytium leading to an increased uterine activity [18]. Currently, nifedipine, which directly blocks the calcium channel, would be in fact the first choice tocolytic agent to postpone delivery [2, 19]. Nifedipine is reported to be highly effective [2]; however, no placebo-controlled trials were performed and nifedipine is not registered for use as a tocolytic agent [20]. In some rare cases, serious maternal side effects are reported due to overdosage of nifedipine [21]. Therefore, more evidence is needed about the efficacy and safety of this drug for the inhibition of labor.

The aims of this study are (1) providing further insight into the use of the electrohysterogram (EHG) for monitoring uterine electrical contractions in preterm labor; (2) evaluating the nifedipine-induced changes in PSD peak frequency in women with preterm contractions.

## 2. Material and Methods

Data were collected at the Máxima Medical Center, a tertiary care teaching hospital. The study was approved by the medical ethical committee of the hospital. All participating women signed an informed consent. 

### 2.1. EHG Signal Recording

**Figure 1:**
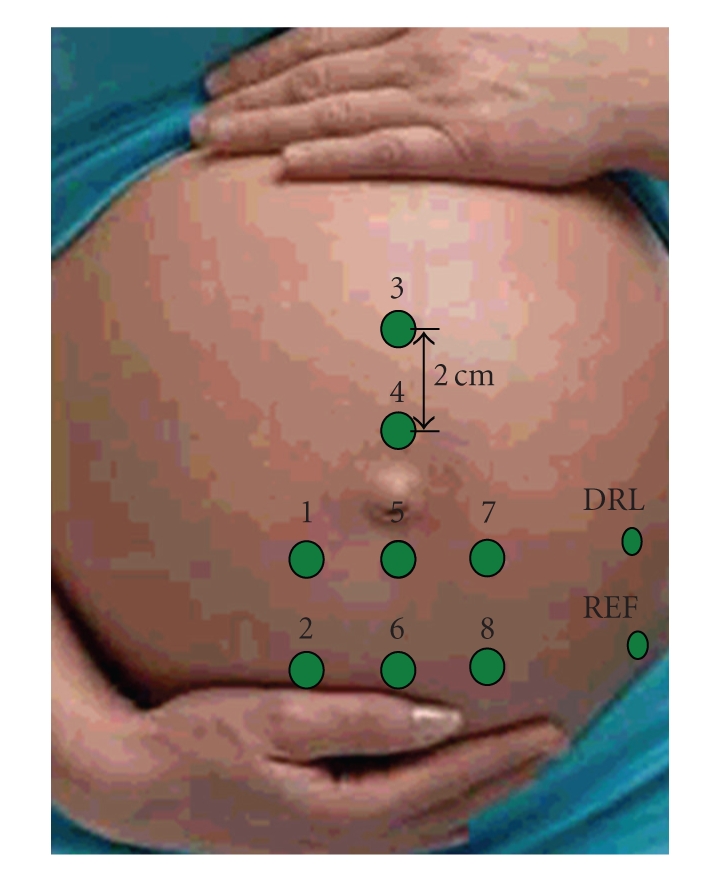
Electrode position and numbering on the abdomen. Prior to electrode positioning, the half length of the uterus was measured. The interelectrode distance was 2 cm for all electrodes. Ref = reference electrode, DRL = ground electrode.

The EHG was recorded by eight disposable contact Ag-AgCl electrodes (2 cm interelectrode distance) placed on the abdomen as shown in Figure 1. Prior to electrode attachment the skin was prepared by gentle rubbing with fine abrasive paper. A reference electrode was placed on the left hip. In order to obtain an efficient rejection of the electromagnetic interference, a driven-right-leg ground electrode and actively shielded cables were employed. The EHG signals were recorded and digitized at 1000 Hz, 20 bit resolution, by an M-PAQ amplifier (Maastricht Instruments Ltd., the Netherlands); a 16-channel system for physiological measurements with programmable gain and sampling frequency. 

All included patients received nifedipine. For each patient, the EHG was recorded twice, before and after giving nifedipine. The period of each recording varied between 20 and 100 minutes per patient. A conventional tocogram was simultaneously recorded for medical prescription. The use of the tocogram was not specifically required for the study, but was employed for confirming the EHG contraction detection.

### 2.2. EHG Signal Processing

In order to improve the signal quality, seven bipolar signals were obtained by subtraction of the signal recorded at electrode five (Figure 1). The recorded signals where then visually inspected to assess the signal quality. If judged of good quality, all seven channels were used for further processing.

Additional noise due to respiration, maternal electrocardiogram, and skeletal electromyogram caused by abdominal muscle contraction was removed by band-pass filtering between 0.34 Hz and 1 Hz [16]. To this end, a four-order band-pass Butterworth filter was employed. The signal was then downsampled up to 20 Hz. The down-sampling was made possible by the low frequency content of the signal and reduced significantly the computational time. Contraction bursts of EHG activity were detected on the EHG signal by the method described in [22] and validated by comparison with the simultaneously recorded tocogram. 

Similarly to many previous studies on the prediction of pre-term labor by electrohysterography, the power spectral density (PSD) was calculated on each bipolar signal by computing the Fast Fourier Transform (FFT) [8, 12, 16, 18]. Only signal segments within the detected EHG electrical contraction burst were used and no information derived from the quiescent period between contractions was analyzed. 

The PSD was separately calculated for three contraction bursts during the first period of measurement, before treatment, and three contraction bursts during the second period of measurement, after treatment. The frequency at which the PSD was maximum, that is, the peak frequency (*f*_*P*_), was singled-out. For each channel, the values of *f*_*P*_ were averaged over the three recorded bursts of electrical activity. Average and standard deviation of resulting *f*_*P*_ for the different periods of recording were then analyzed and compared.

### 2.3. Study Population

The study protocol has been carried out in addition to the general used procedures for threatened preterm labor.

Women admitted to the hospital for spontaneous preterm contractions, at risk for extreme preterm delivery, that is, gestational age between 24th and 32nd weeks, were included in the study. 

Exclusion criteria were age under 18 years, body mass index above 30, cervical dilatation over 3 cm. Additionally were excluded from the study those pregnancies complicated by pre-eclampsia or HELLP syndrome, placenta praevia, fetal anomalies or maternal trauma precipitating the symptoms. 

Immediately after admission to the hospital, patients were assessed by a clinician in order to evaluate the risk of preterm delivery. The criteria for preterm labor were regular contractions at a rate of 4 in 20 minutes or 8 in 1 hour with at least one of the following: progressive effacement or dilatation of the cervix over time or dilatation of the cervix greater than or equal to 2.0 cm. If they were judged to be at risk, they were treated according to the hospital guidelines following the national guideline for tocolysis of the Dutch Association for Obstetrics and Gynecology [23].

Three primiparous and five multiparous women attending the hospital for preterm contractions underwent multichannel EHG recording. The mean gestational age on admission was 28.2 ± 2.8 weeks. 

All singleton pregnancies received nifedipine oros 120 mg daily for the first 48 hours. After that, the dosage was lowered gradually over a 4-day period.

The two twin gestations received nifedipine oros 90 mg as the maximum dosage in order to decrease the risk of acute lung oedema [24]. Before giving nifedipine oros, in all pregnancies a load up dose with nifedipine 20 or 40 mg was given. betamethasone for fetal lung maturation was given to 7 patients, in the other patient betamethasone was already completed in the referring hospital. 

Three different analyses were done by comparing the PSD peak frequency among different patient groups. The first comparison (three subjects, Group 1) was done between the value of *f*_*P*_ derived from EHG signals recorded before (Subgroup 1.1) and 15 minutes after administration of nifedipine (Subgroup 1.2). For those patients who already started nifedepine treatment in the referring hospital before the first EHG measurement session (three subjects, Group2), the comparison was done between the first measurement, that is, within 24 hours after starting nifedipine oros 9 (Subgroup 2.1), and one day after finishing the tocolytic treatment (Subgroup 2.2). After the effects of betamethasone were expected to be occurred, that is, 48 hours after the first injection, tocolytic treatment was finished in a cutback plan over a 4-day period. The third analysis included all patients (Group3) and aimed at a retrospective comparison of the *f*_*P*_ measured within 24 hours after starting treatment with nifedipine on women who delivered within one week (Subgroup 3.1) and the *f*_*P*_ measured within 24 hours after starting treatment with nifedipine on women who delivered after more than one week (removed: from the EHG measurement) (Subgroup 3.2).

Of the included patients, five women were diagnosed with lower urinary tract infection at 2–4 days before attending the hospital and four of them were still being treated with antibiotics when nifedipine was given.

 Patient's characteristics are summarized in Table 1.

### 2.4. Statistical Analysis

Sigma-Stat software (SPPS Inc, Chicago, IL) was implemented for statistical comparison of groups. The PSD peak frequencies in all channels for every single patient and all separate bursts were considered for analysis.

Statistical analysis was performed using a 2-tailed *t*-student. A *P *value of less than  .05 was considered statistically significant.

## 3. Results

**Figure 2:**
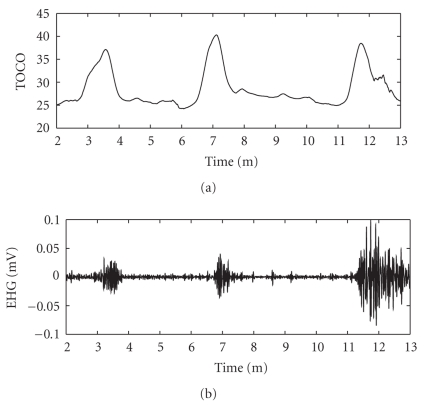
Tocographic and EHG recording of a patient with preterm uterine contractions. The figure shows an example of the temporal correlation between the uterine electrical and mechanical activity.

In all patients, the uterine contractions detected by the EHG signal analysis corresponded to the uterine mechanical activity as recorded by tocodynamometer. In Figure 2 an example of these typical recordings is given for one patient.

In general, the peak frequency as well as the uterine activity decreased in all patients after treatment with nifedipine (Table 2).

**Figure 3:**
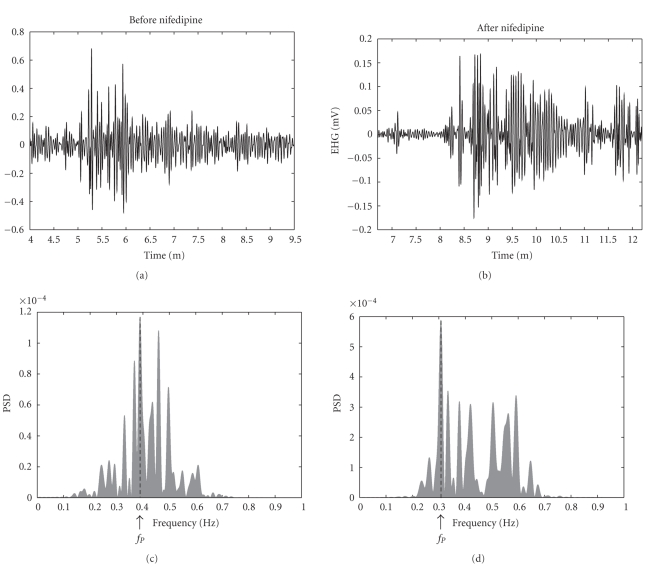
Example of preprocessed EHG signal (upper plot) before (left) and after (right) nifedipine treatment. The corresponding power spectral densities before (left) and after (right) nifedipine treatment are depicted in the bottom part of the figure with an indication of the peak frequency *f*_*P*_.

In three patients (Group 1), the EHG signal was recorded before (62 bursts analyzed in total) and 15 minutes (time expected that effect of nifedipine will occur) after administration of nifedipine (53 bursts analyzed in total). As shown in Table 2 (group 1), averaging the results in these three patients, a significant 7.2% (*P* < .05) decrease in PSD peak frequency was observed (Figure 3). 

In three other patients (Group 2), a comparison was made between the EHG signal recorded within 24 hours after starting treatment with nifedipine (56 bursts analyzed in total) and the EHG signal recorded 1 day after finishing tocolytic treatment after the 6-day period of treatment (63 bursts analyzed in total). Also in this case, a significant (*P* < .05) decrease in PSD peak frequency was observed in all patients (Table 2, Group 2). On average, the PSD peak frequency decreased of 6.7%.

Finally, from the eight patients in which the EHG was recorded (Group 3), three patients delivered within 1 week after treatment with nifedipine (Subgroup 3.2). Frequency analysis of the signals recorded from these patients (3 patients, 56 bursts analyzed in total), to those patients delivering after more than 1 week (Subgroup 3.2, 5 patients, 98 bursts analyzed in total), showed, on average, a higher *f*_*P*_ for Subgroup 3.1 as shown in Table 2. However, the significance of this difference was poor (*P* = .46).

## 4. Discussion

The devices and methods currently used for predicting preterm delivery, such as vaginal examination, tocodynamometer, fetal fibronectin, and ultrasound measurement of cervical length, do not provide accurate diagnosis or prediction of preterm labor [25]. Intrauterine pressure catheters are reliable but limited by their invasiveness and the need for ruptured membranes. External uterine monitors, such as tocodynamometers, are uncomfortable, inaccurate, and depend on the personal interpretation of the examiner. Biological tests, such as fetal fibronectin, can be used as a prognostic marker, although with poor positive predictive values [5]. Even cervical length changes may not be an accurate indicator of true labor, as a large percentage of women with established cervical change do not deliver preterm even without tocolytic treatment [26]. 

As current methods are not capable of discriminating contractions, most obstetricians either treat all patients having preterm contractions or wait for cervical change. Delay in the diagnosis of preterm labor may result in a lower efficacy of tocolytic drugs [27, 28], while giving tocolysis to all patients exhibiting uterine activity is not free of risks for mother and fetus [21, 28, 29]. 

Uterine contractions are the direct consequence of the generation and propagation of electrical activity at the myometrium [6, 7]. It has been demonstrated that the EHG signal recorded on the skin surface is representative of the electrical activity initiating the mechanical contraction of the uterus [7]. Being a measure of the first cause of a contraction, the EHG signal is potentially the best predictor of delivery. Furthermore, it is noninvasively and can therefore be employed for both singleton and multiple gestations during pregnancy and delivery.

For the prediction of labor by electrohysterography previous studies mainly focused on the analysis of the frequency properties of the EHG signal [8, 9, 14, 15].

There is general agreement that the peak frequency derived from the PSD of the signal increases as the measurement-to-delivery time interval decreases [13, 16]. In particular, Buhimschi et al. [12] detected a significant shift from low to high PSD peak frequency in nonlaboring preterm patients as compared to laboring preterm patients. Doret et al. [11] performed a study in rats and showed the PSD peak frequency to be the earliest change to occur in the EHG signal as preterm delivery approached.

The shift of the EHG signal spectral content to higher frequencies can be explained by the underlying physiology. The frequency of action potentials within a burst is, in fact, a direct measure of the rate of the depolarization/repolarization process in the myometrial cells, a process governed by calcium ion-influx across ion channels [30]. When modifications in the ion channels of the myometrial cell plasma membrane initiate labor, the uterus becomes more excitable [30, 31], the signal propagation distance and contraction strength increase, and, as a result, higher frequencies within bursts of activity [13, 16] are expected.

Nifedipine is a calcium channel blocker. Therefore, it can be expected to act directly on the propagation of uterine activity. Lower frequencies within the EHG bursts after tocolytics can be associated to a lower level of electrical activity propagation and, ultimately, to the effectiveness of the treatment. The majority of tocolytic agents attempt to paralyze the myometrium, without addressing the root stimulus of preterm labor in a cause-specific way. Conversely, nifedipine acts by inhibiting the influx of calcium ions through the cell membrane and the release of intracellular calcium from the sarcoplasmatic reticulum. As a result, a decrease in intracellular free calcium occurs and leads to the inhibition of myosin light-chain kinase-mediated phosphorylation, which is calcium-dependant, and inducing a relaxation of the myometrium [32].

Moreover, nifedipine can be a preferable treatment for patients at risk for preterm delivery because it can be administered by oral route at any gestational age and severe side effects are reported to be very rare [20].

In the present study, the PSD peak frequency decreased in all patients after treatment with nifedipine, also for those patients close to delivery. The same results were obtained by analyzing the EHG signal of patients who were recorded within 24 hours of first treatment compared to their EHG signal after end of treatment. 

A disadvantage of this study is that there is no control group. However, all of the patients we included in the study were at high risk for delivering very preterm. 

The PSD *f*_*P*_ tendency highlighted in the present study suggests that nifedipine can be an effective tocolytic agent for inhibiting electrical activity propagation and therefore the effectiveness of uterine contraction when occurring preterm. In this case, the ultimate goal is in fact delaying the delivery in order to allow medical intervention or, in the most desirable case, to reach the term of pregnancy.

In the present study, three patients delivered in 1 week, even if they had been treated with nifedipine. All these women had an asymptomatic urinary tract infection. It is well established that treatment with antibiotics is associated with prevention of preterm birth [33]. In our study, four of the five patients were treated with antibiotics and two of them did not deliver within one week. The effectiveness of tocolytics in the presence of urinary tract infections needs further to be defined.

The reason of the failure of tocolytics in preventing preterm delivery is still an unsolved issue for gynecologists. However, it has been suggested that the effectiveness of tocolytics is highly dependent on early initiation of the therapy. Therefore, timely recognition of the process leading to the delivery is fundamental. Garfield et al. [18] and Linhart et al. [26] demonstrated, in fact, that parturition is a two-step process consisting of a conditioning (preparatory) phase, followed by active labor. During the conditioning phase, there is a progression of uterine contractility from an inactive to an active state. In the myometrium, the preparatory process involves changes in transduction mechanisms and the synthesis of several new proteins. At some point of the conditioning step, the process becomes irreversible and leads to active labor and, ultimately, to delivery [18]. It is known that agents used to intervene in labor may be of greatest value during the preparatory phase in the progression to delivery [1]. Therefore, it could be suggested that the women in this study who failed to respond to therapy were treated too late and that during the EHG recording, an irreversible change to the active phase of labor might already have occurred. 

On average, the women who did not respond to the therapy and delivered within one week from the measurement showed a higher *f*_*P*_ than the other patients. However, not all patients within one week from delivery showed higher *f*_*P*_ than the rest; finding a cut-off value for an accurate classification of the two populations was therefore not possible. Due to the limited size of the study population, these preliminary results, even if obtained by analyzing a large number of bursts, might be affected by the different treatment-to-measurement time. 

We showed that the PSD peak frequency decreased in all women after treatment with nifedipine, although, due to the small number of women in the study and variable times of EHG recording (in five of the eight women, EHG recordings could only be made after the start of nifedipine treatment), this could not be linked with a significant increase in time to delivery. 

Previous studies on a more extended database on animals and women not treated with tocolytics concluded that the peak frequency derived from the PSD of the EHG signal increases as the measurement-to-delivery interval decreases [17]. Noticeably, these conclusions were also mostly derived by mean differences for all the patients. However, ultimately, due to a poor significance of the difference, a cut-off value of peak frequency has not been found yet. Therefore, further studies are required in order to develop methods that can reliably differentiate the uterine status of labor. Longitudinal studies in pregnant women could support the determination of the EHG properties related to the electrophysiological process leading to preterm labor. Further research on the differentiation of the uterine electrical activity in the non-prepared state before labor and the active state of labor would be necessary; objective criteria to evaluate the state of the uterine preparedness for labor can in fact improve management of preterm labor.

## 5. Conclusion

In this study, the efficacy of nifedipine as a tocolytic drug on the uterine activity was evaluated by monitoring and analysis of the EHG signal. Treatment with nifedipine leads to a statistically significant shift to lower PDS peak frequency in the EHG. As the PDS peak frequency is expected to rise when delivery approaches, from these preliminary results, it could be concluded that nifedipine is an effective tocolytic agent for suppressing preterm contractions. However, further studies are needed to relate the decrease in the myometrium electrical activity to the physiological effects leading to the effectiveness of the tocolitic treatment.

Even with accurate tocolysis, some patients do not respond to therapy and preterm delivery cannot not be inhibited. In these patients, an irreversible change to the active phase of labor has probably occurred. As tocolytic agents may be more effective during certain periods in the progression to delivery, it is important to develop methods that could reliably diagnose the risk of preterm birth at early stages. 

This study confirmed that the EHG signal analysis is a reliable technique for non-invasive monitoring the uterine contraction and has great potential for predicting labor, especially for patients at high risk for preterm delivery. However, considering also the previous literature on the topic, our results suggest that the analysis of the EHG signal frequency alone may not provide a universal cut-off value for assessing whether the irreversible process leading to labor has already started or not. Since the spreading of the electrical activity through the myometrium is the first cause of labor, the analysis of the EHG signal propagation, for example, in terms of direction and velocity [34–36], might provide an important contribution to the development of methods that can objectively evaluate the state of the uterine preparedness for labor and to identify the patients at risk for preterm labor.

##  Contribution to Authorship

Maartje P. G. C. Vinken worked on conception and design of data, acquisition of data, and analysis and interpretation of data; C. Rabotti on analysis and interpretation of data, critical revision of article; M. Mischi on critical revision of article and statistical analysis; Judith O. E. H. van and G. S. Oei on the critical revision of article.

## Figures and Tables

**Table 1:** Study population characteristics.

Pt	Age	GA^1^ at first EHG recording	Pregnancy	BMI^2^	Cervical dilatation (cm)	Cervical length (cm)	PPROM^3^	UTI^4^	Nifedipine-recording- interval (hours)	Delivery < 1wk
1	29	24 + 1	Singleton	22.3	1	3.1	No	No	4	No
2	34	28 + 0	Singleton	27.8	1	2.3	No	No	9	No
3	32	25 + 4	Singleton	28.0	0	1.6	No	No	10	No
4	29	30 + 4	Twin	22.5	1	1.9	No	Yes	8	Yes
5	29	31 + 2	Twin	21.6	1	2.0	Yes	Yes	5	Yes
6	28	29 + 5	Twin	27.3	1	2.2	No	Yes	0	No
7	34	25 + 6	Twin	23.4	2	2.6	Yes	Yes	0	No
8	34	31 + 1	Singleton	25.1	1	ntb	No	Yes	0	Yes

^1^ = gestational age

^2^ = body mas index

^3^ = preterm prelabor rupture of membranes

^4^ = urinary tract infection.

**Table 2:** Shift of PSD peak frequency.

Group 1^1^	Before nifedipine (Subgroup 1.1)	15 minutes after nifedipine (subgroup 1.2)	*P*-value
PDS peak frequency (Hz)	0.367 ± 0.061	0.340 ± 0.040	
	0.415 ± 0.046	0.375 ± 0.051	
	0.424 ± 0.079	0.405 ± 0.021	
*Mean *	0.402 ± 0.025	0. 373 ± 0.026	.043*

Group 2^2^	Within 24 hours after starting nifedipine (subgroup 2.1)	1 day after finishing nifedipine (subgroup 2.2)	*P*-value

PDS peak frequency (Hz)	0.390 ± 0.035	0.371 ± 0.058	
	0.436 ± 0.046	0.403 ± 0.074	
	0.434 ± 0.047	0.403 ± 0.041	
*Mean*	0.42 ± 0.021	0.392 ± 0.015	.024*

Group 3^3^	Patients delivering after 1 week after start nifedipine (subgroup 3.1)	Patients delivering within 1 week after start nifedipine (subgroup 3.2)	*P*-value

PDS peak frequency (Hz)	0.340 ± 0.040	0.407 ± 0.065	
	0.375 ± 0.051	0.380 ± 0.055	
	0.405 ± 0.021	0.434 ± 0.047	
	0.436 ± 0.046		
	0.390 ± 0.035		
*Mean*	0.389 ± 0.031	0.407 ± 0.022	.458**

^1^ = patient 1–3 (Table 1)

^2^ = patient 4–6 (Table 1)

^3^ = patient 1–8 (Table 1)

*calculation by 2-tailed paired student *t* test

**calculation by 2-tailed unpaired student *t*-test.
